# Development and Effect of a Fall Prevention Program Based on King’s Theory of Goal Attainment in Long-Term Care Hospitals: An Experimental Study

**DOI:** 10.3390/healthcare9060715

**Published:** 2021-06-10

**Authors:** Bom-Mi Park

**Affiliations:** Department of Nursing, Konkuk University Glocal Campus, Chungju-si 27478, Korea; spring0317@kku.ac.kr; Tel.: +82-43-840-3960

**Keywords:** accidental falls, aged, goal, long-term care, patients

## Abstract

A fall prevention program based on King’s goal attainment theory was developed to verify its effect on those in long-term care hospitals. The experiment was conducted at K Long-Term Care Hospital in S city for eight weeks. The study employed 57 elderly patients and 58 nurses. The program comprised an individual training conducted in a ward and hospital room for 20–30 min and a group training held in a conference room for 60 min. Significance levels were analyzed at *p* < 0.05 via frequency analysis, descriptive statistics, independent sample *t*-test, χ^2^-test, Mann–Whitney’s U test, Wilcoxon code rank test, and Cronbach’s α, and the clinical trial number was KCT0005908. In the patient intervention group, fall prevention behavior and knowledge increased, and the fear of falling decreased. Fall prevention behavior and knowledge increased in the nurse intervention group. Patient and nurse interaction satisfaction also increased. In contrast, the number of falls and nurses’ burden did not decrease. The fall prevention program was verified via the interaction of personal, interpersonal, and social systems. Thus, the patient’s fear of falling was reduced. Moreover, the program was effective for the fall knowledge, interaction satisfaction, and fall prevention behavior of both the patient and nurse.

## 1. Introduction

In Korea, the elderly population aged 65 and above is expected to rapidly increase from 14.9% in 2019 to 20.3% in 2025 and 46.5% in 2067. Moreover, the number of elderly households aged 65 and above would increase 2.8-fold from 3,998,000 households (20.4%) in 2017 to 11,058,000 (49.9%) in 2047 [1,2]. The average annual medical expense per person (>65 years old) is 4,910,000 won, which is about three times higher than the annual average medical expenses of 1,680,000 won, per applied population in 2019 [3].

Falls are a frequent problem observed in elderly patients in long-term care hospitals [4,5]. They can cause psychological (e.g., fear of falling, anxiety, pain, and depression) and physical problems [4]. Severe cases may lead to death [6]. Falls are a serious problem; they are the largest hospital accident category [7]. The World Health Organization (WHO) reported that 646,000 people die each year from falls worldwide, and falls are most commonly observed in the elderly over age 65 [8]. Falling experience in the elderly is a serious sequela that lowers physical function, induces loss of daily life independence, and limits physical, psychological, and social activities, ultimately lowering life quality [9]. Further, fear of falling may reduce daily activities and weaken muscles, leading to an increased risk of falling [10]. Accordingly, the WHO reported that fall prevention programs based on environmental factors of hospitals are effective for hospitalized patients with a high risk of or previous experiences of falling [11]. Thus, various measures to standardize nursing practices to prevent falls [12], improve work processes, and reduce environmental restrictions [13] are necessary.

Intrinsic factors such as weak lower extremities, falling experience, lack of gain/balance, and visual field defects and extrinsic factors such as insufficient lighting high beds and chairs. Inadequate assistive devices and improperly fitted shoes are highly corrected with patient falls [14]. In particular, elderly patients who are admitted to long-term care hospitals have difficulties in moving due to diseases that decrease cognitive function, such as dementia or stroke [15]. Therefore, nurses are responsible for assessing fall risk factors for elderly patients with high risk of falls and providing fall prevention care accordingly [16].

Nurses have the greatest effect on reducing the number of falls among patients [17] since they directly interact with patients the most [14]. Nursing goals are achieved via patient–nurse interactions; thus, effective interaction is an important tool for patient treatment [18] and an essential condition for nurses to establish a therapeutic relationship with patients [19]. Therefore, nurses must interact with patients and induce fall prevention behaviors through repetitive fall prevention education programs, considering individual circumstances, diseases, and medication status [9].

King’s goal attainment theory defines a process of interaction where patients and nurses acknowledge each other, set goals, and agree on how to achieve them in fulfilling exchanges between patients and nurses [20]. The theory pivots on respect for patients. It is a patient–nurse relationship that values information exchange, goal setting, and patient treatment; thus, it requires a positive correlation between trust and patient satisfaction [20,21]. Moreover, it must include interactions that describe patient–nurse values and needs [20,21]. The theory posits a high probability of achieving goals when patients and nurses interact and set goals together [20]. Such interactions allow patients to assume responsibility for and actively participate in the proposed treatment for positive changes. Thus, the goal attainment theory is a health strategy in nursing patients [22].

Recent studies on fall prevention programs include fall education [23,24], exercise and pain control [25], and motivation and group discussions [26]. Goal attainment theory has effectively reduced the number of falls in elderly patients at a high risk of falling [27]. However, 31% of falls and 47% of falls with injuries occur among patients [28]. Moreover, those who overestimate and are not aware of their functional capacities have a high risk of falling [29,30]. Studies have also shown that 12.3% of patients overestimate and forget their functional levels [31]. In contrast, elderly patients at high risk of falling are more aware of and cautious about the risk of falls [32]. Thus, a fall prevention program based on King’s goal attainment theory where patients better understand their conditions [33] and set behavior modification goals [34] via interactions with nurses is essential.

Many studies aim to prevent falls. However, only a few incorporate fall theory and demonstrations, medication education, environmental management, motivation, and individual repetitive education tailored to the personal circumstances of the elderly. Therefore, this study developed a fall prevention program where patients and nurses set goals together and actively participate. These observations provide the basis for an effective nursing intervention program. Specifically, the study evaluates the program’s effects on (1) the fear of falling, fall knowledge, fall prevention behavior, and the number of falls among the patient groups; (2) the burden of falling, fall knowledge, and fall prevention behavior among nurse groups; and (3) the interaction satisfaction between patients and nurses.

The conceptual framework was established based on King’s goal attainment theory from a review of the relevant literature. First, perception is an individual’s experience and image of reality, and an individual’s perception and judgment translate to actions and responses. Second, current obstacles and problems, the setting of mutual goals, and seeking and agreeing on how to achieve these goals are assessed via interactions. Third, interactions through personal, interpersonal, and social systems lead to the achievement of goals [35,36].

Patients and nurses acted via the perception and judgment processes. Actions include nurses’ suggestions for patients to participate in the program and patients’ positive reactions to participation. Response refers to their agreeing to participate in the program.

In the interaction system, disturbances or problematic factors during the program are identified by assessing factors related to falls, interest in fall prevention, and relationships with the surrounding environment. Mutual goal setting is performed by patients and nurses who explore the situation together and share information. Exploration and agreement on how to achieve goals comprise a discussion of the program methods. Individual education, group education, individual counseling, and individual activities were performed to achieve the goals.

Transaction refers to the interaction between patients and nurses through personal, interpersonal, and social systems to achieve the goal of reducing the total number of falls by reducing the fear of falling among patients and the burden of falling on the nurses while increasing fall knowledge, interaction satisfaction, and fall prevention behaviors.

In this study, it is expected that a fall prevention program based on King’s goal achievement theory would reduce patients’ number of falls and fear of falling, and increase fall knowledge, fall prevention behavior, and interaction satisfaction. In addition, it is expected that nurses’ burden of falling would be reduced, and fall knowledge, fall prevention behaviors and interaction satisfaction would be increased.

## 2. Methods

### 2.1. Fall Prevention Program Based on King’s Goal Achievement Theory

#### 2.1.1. Fall Prevention Program Contents

**Figure 1:**
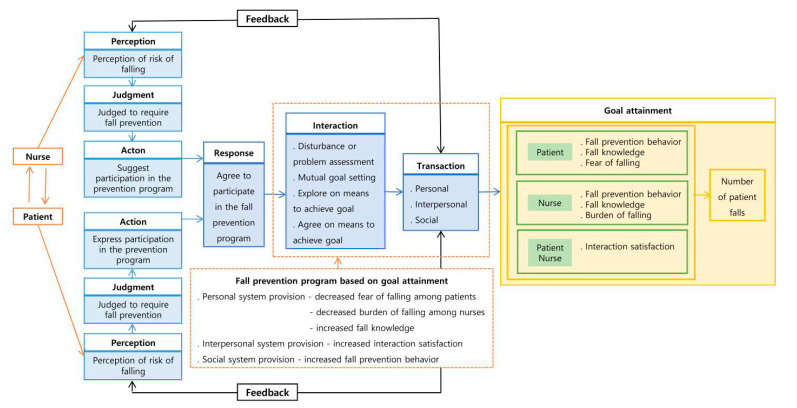
Conceptual framework of a fall prevention program on King’s goal attainment theory.

This study employed a goal attainment theory-based intervention program, developed via a review of the literature on fall-related intervention programs for patients and nurses. Figure 1 illustrates the relationship between the concepts of this study. The program was offered once a week for eight weeks (comprising individual education, group education for nurses only, and emotional support) to establish a mutual goal between the researchers and both patients and nurses, respectively. Furthermore, the program comprised various contents such as fall prevention education and demonstration, medication education, environmental management, motivation, and repetitive individual education. The assessed outcome variables included fear of falling, the burden of falling, fall knowledge, interaction satisfaction, fall prevention behavior, and the number of patient falls (Table 1).

The personal system comprises the recognition of the importance of fall prevention, personal growth, and development during the program. In this system, problems related to falls among patients were assessed, and knowledge and information were provided via individual education and counseling to reduce the fear of falling. Thus, to reduce the burden of falling on nurses, problems related to falls among nurses were assessed, and related knowledge and information were provided through individual education and counseling. Moreover, knowledge and information were provided to patients and nurses to improve their fall knowledge, and guidelines and handouts on fall prevention were provided to improve fall knowledge cognition.

The interpersonal system was characterized as nurses and patients setting goals together and improving satisfaction with interaction via communication. Mutual goals were set, and motivation was provided to improve interaction satisfaction among patients and nurses and assess barriers to fall prevention. Further, related knowledge and information were provided. Education and demonstrations of fall prevention guidelines were provided, after which participants were required to reiterate their understanding. Patients and nurses evaluated the fall prevention checklist, as education on fall prevention and medication was provided.

Social systems are defined by repetitive fall education systems for patients and nurses and improvement of fall prevention behaviors via social support from researchers and nurses. Fall prevention education was provided to patients and nurses to improve their abilities and behaviors. Physiotherapists and nurses in charge of patient safety provided fall prevention education. Counseling and support on challenges and concerns about falls were provided to patients, and continuous participation was encouraged for fall prevention to motivate and provide emotional support to nurses to induce fall prevention behaviors.

Fall prevention guidelines and checklists were created by selecting necessary items from the Fall Prevention Guideline by the US Health Care Improvement Organization [37], certification survey standard collection of long-term care hospitals by the Korea Institute for Healthcare Accreditation [38], and safety assurance activities of the 1st Comprehensive Patient Safety Plan proposed by the Ministry of Health and Welfare [39] to suit the characteristics of long-term care hospitals. The items were then reviewed and verified by a member of the certification evaluation survey of long-term care hospitals, a rehabilitation medicine specialist, and two physiotherapists. The fall prevention program was reviewed and verified by a nursing college professor, an individual in charge of patient safety, and six head nurses.

A. Individual education

The patients were vacant from the hospital room for 30–120 min in the morning and afternoon for rehabilitation treatment. Therefore, each patient’s schedule was checked with the patient or caregiver to organize individual patient schedules before the study. Individual training was provided in the hospital room for 20–30 min before and after the rehabilitation treatment.

Individual education was provided once a week for eight weeks. Six (two) sessions were conducted by the researcher (nurse in charge). Individual education to nurses was provided by the researcher once a week for seven weeks. The nurses worked in three shifts. Thus, their schedule was organized for eight weeks by checking the researcher’s shift schedule. For nurses with day, evening, and night shifts, the researcher provided education after the shift, before the shift or in the evening, and in the morning after shift, respectively, for 20–30 min.

Individual education for nurses was provided seven times. The goal was to provide individual education to ten patients and ten nurses every day. However, in cases where the patients and nurses were not available due to schedule conflicts, individual education was provided on weekends (Table 1).

B. Group education

Group education was provided only to nurses in the evening of the second week for one session of 60 min in a conference room of the hospital, where demonstrations were provided. The session was provided by a nurse in charge of patient safety and two rehabilitation therapists. After the education and demonstrations for fall prevention by therapists, two nurses teamed up to practice. Chairs and braces were prepared in advance such that 19 nurses could practice immediately after training. Individual feedback was provided to each nurse team by a rehabilitation therapist. For six nurses with evening shifts and five nurses who could not participate, the researcher was permitted to film the demonstrations by the rehabilitation therapists to provide individual education. In the wards, videos were shown individually to the nurses, and they practiced with chairs and braces in pairs with the researcher.

C. Individual counseling

In the first week of individual counseling, fall-related problems were checked by nurses, and sessions were held weekly during individual education. Challenges and concerns about fall behaviors were mostly consulted and, in weeks 5 and 6, the interaction importance was emphasized for encouragement and support.

D. Individual activities

In weeks 5 and 6, individual activities were conducted for assigned patient–nurse interactions. Such interactions without the researcher are vital for fall prevention behaviors beyond the 8-week period. Thus, the nurses who received individual education from the researcher provided the same education to the assigned patients.

In week 5, the nurses were asked to practice the 5A (Ask-Advise-Assess-Assist-Arrange) method of the Clinical Guidelines for Smoking Cessation, published by the US Public Health Service [40], on the assigned patients via effective communication. First, the nurses “asked” the patients questions regarding falls. Second, the nurses provided clear information and brief and personalized “advice” on the risk factors of falls. Third, personal relevance to the risk and prevention of all patients was “assessed.” Fourth, confidence in planning for change, acquiring behavioral skills, and success in preventing falls was provided whenever “assistance” was needed. Finally, the fall prevention behavior of patients was supported and encouraged in the “arrange” stage.

In week 6, the efficacy and side effects of medications were explained by the nurses to the assigned patients; they were educated on their importance and danger. Further, the nurses provided fall prevention education and demonstrations to assigned patients, in which patients directed their questions to the nurses. A grape sticker was provided if no fall was observed every week, and after eight weeks, a gift (a towel set) was provided to the patients who acquired a grape sticker for all eight weeks (Table 1).

#### 2.1.2. Weekly Themes and Goals of the Fall Prevention Program 

The theme of the first week was “reducing fear and burden of falling,” and the goal was “identifying fall problems and setting goals.” The patients and nurses shared their fear and burden of falling based on direct and indirect experiences, and they sympathized with the necessity of preventing falls and setting mutual goals.

The theme of weeks 2 to 4 was “improvement of fall knowledge,” and the goals were “understanding and practicing fall prevention education” and “solving problems after individualized fall prevention education.” Education on fall prevention guidelines and checklists was conducted individually and in groups.

The theme of weeks 5 and 6 was “interaction,” with a goal of “promoting fall prevention through interaction.” The nurses provided individual education on fall prevention, efficacy, and side effects of medications via the 5As based on the education they received from the researcher. The patients were educated to be aware of hypertension, diabetes, dysuria, and anti-psychotics, which are high-risk factors for falls.

The theme of weeks 7 and 8 was “improvement of fall prevention behavior,” with the goal of “improved execution through individualized fall education.” The researchers held a discussion with patients and nurses and compared well-executed fall prevention behaviors to those otherwise to improve the practice of fall prevention behaviors.

### 2.2. Verification of the Effects of the Fall Prevention Program Based on King’s Goal Attainment Theory

#### 2.2.1. Design

The non-equivalent control group (pre- and post-test study) was conducted at K long-term care hospital, located in S city, for eight weeks from 12 July 2019 to 5 September 2019. The study comprised 57 elderly patients (27 and 30 in the intervention and control groups, respectively) and 58 nurses (28 and 30 in the intervention and control groups, respectively).

In the control group, the conventional fall prevention program (patient fall prevention education after hospitalization, periodic evaluation of patient falls, quarterly multidisciplinary team meetings on fall, fall prevention announcements before bedtime, notification announcements in cases of falls, fall prevention rounds, posting status on fall accidents, and improvement plans) were provided.

#### 2.2.2. Participants and Sampling Method

In this study, patients and nurses were recruited from elderly long-term care hospitals in Seoul, South Korea, with more than 300 beds. The study was explained to the participants, and those who wished to participate in the program after the explanation were selected. Thus, to prevent the diffusion of treatments in the control and intervention groups, the floor separation intervention study by Krauss et al. [41] was used as a reference.

The second (fourth) and third (fifth) floors were grouped together, and a nurse randomly allocated the floors to intervention and control groups in blinded settings. Within the hospital, patients are restricted from moving without caregivers or family members, who can only move to the first floor, patient floors, and rehabilitation treatment floor with an access card. Thus, a slight possibility of treatment diffusion existed. As the patients underwent 1:1 rehabilitation treatment with a therapist, conversations between patients were challenging. The nurses also conducted their shifts in the corresponding ward with little interaction between different floors.

**Figure 2:**
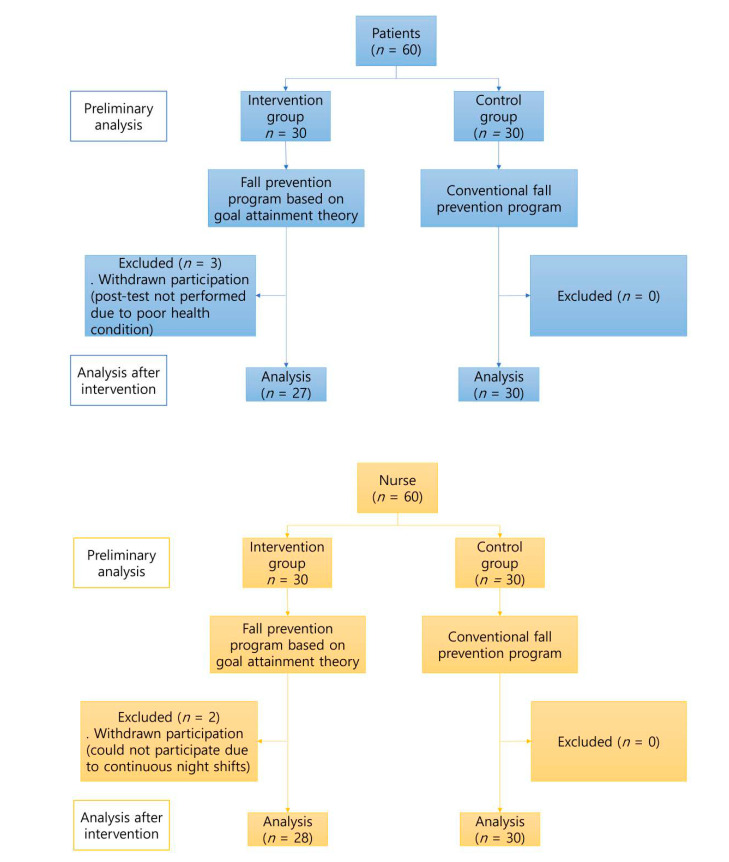
Flow chart of the study.

The minimum sample size was calculated using the G*power 3.1(Kiel University, Kiel, Germany.) [42]. It was based on the effect size of (*d*) = 1.20, following a similar study by Jung and Kim [43], where the goal attainment theory was applied. With a significance level (α) = 0.05, power (1-β) = 0.80, and effect size (*d*) = 0.08, the minimum sample size for both groups was 21 and, given possible withdrawals, 30, which is 1.4 times greater than the required size selected for each group. Three patients who were unwilling or could not answer the questionnaire due to poor health were excluded; thus, 27 and 30 patients were included in the intervention and control groups, respectively. Two nurses on night shifts could not continue their part in the study and, thus, were excluded. Therefore, 28 and 30 nurses were included in the intervention and control groups, respectively. Figure 2 presents a flowchart of the study.

Patient selection criteria included the following: (a) those who understood the purpose of the study and agreed to participate, (b) those whose legal guardian consented to the study, and (c) those who communicated with nurses and complete the survey questionnaires. Those expected to be discharged during the intervention period were excluded. The selection criteria for nurses were those who understood the purpose of this study and agreed to participate.

Data collections was conducted by 2 researchers and the collection was conducted at the start of the intervention and 8 weeks after the intervention.

#### 2.2.3. Research Tools

##### Patients

*Fear of falling*. To measure the fear of falling, a tool developed by Tinetti et al. [44], adapted by Jang [45], and modified by Kim [46] was used. The tool consists of 10 items, and the total score ranges from 10 to 100 points. A higher score indicated a greater fear of falling. In this study, the mean value was calculated by dividing the total score by the number of items for unity by the other item scores.

*Fall knowledge.* A tool developed by Kim [47] and modified by Kim [48] was used to measure fall knowledge. The total score ranged from 0 to 15, with higher scores indicating greater fall knowledge.

*Interaction satisfaction.* A tool developed by Lim and Kwon [49] and modified by Kim [50] was used to measure interaction satisfaction. It comprised nine items, evaluated on a 5-point Likert scale; a higher score indicated greater interaction satisfaction.

*Fall prevention behavior.* A tool developed by Kim [48] for hospitalized elderly patients was used to assess fall prevention behaviors. The tool comprised 10 questions, evaluated on a 5-point Likert scale, with a higher score indicating a higher level of fall prevention behaviors.

##### Nurses

*Burden of falling.* A tool developed by Kim and Kim [51] was used to measure the burden of falling. This tool comprised 16 items, evaluated on a 4-point Likert scale, with a higher score indicating a greater burden of falling.

*Fall knowledge.* A tool developed by Kim [47] and modified and complemented by Kim and Seo [52] was used to measure fall knowledge. It comprised 16 items, and the total score ranged from 0 to 16 points. A higher point indicated greater fall knowledge.

*Interaction satisfaction.* A tool developed by Lim and Kwon [49] and modified and complemented by Kim [50] was used to measure interaction satisfaction. It comprised nine items, evaluated on a 5-point Likert scale, with a higher score indicating greater interaction satisfaction.

*Fall prevention behavior.* Kim [48] developed a tool for hospitalized elderly patients, modified and complemented by Kim and Seo [52], for nurses in hospitals for the elderly to assess fall prevention behaviors. The tool comprised 13 items, evaluated on a 5-point Likert scale, with a higher score indicating a higher level of fall prevention behaviors.

#### 2.2.4. Data Analysis

The data were analyzed using SPSS 25.0. Frequency analysis on categorical variables assessed the general and disease-related characteristics of patients and general characteristics of nurses. Descriptive statistics were employed for continuous variables. Independent sample *t*-tests and χ^2^ tests were performed to verify the differences between the general and disease-related characteristics of patients and the general characteristics of nurses. Skewness and kurtosis determined whether the data satisfied the normality assumption, and a non-parametric statistical analysis was performed when skewness and kurtosis were less than two and absolute values of seven, respectively. The Mann–Whitney test was conducted to gauge the differences in the number of falls between the groups, and Wilcoxon’s signed-rank test was performed to gauge the difference in the pre- and post-test number of falls. Cronbach’s α assessed the tools’ reliability, and the statistical significance level was *p* < 0.05. An independent sample *t*-test assessed pre-test homogeneity, post-test differences, and differences in the amount of change between the groups for fear of falling, fall knowledge, interaction satisfaction, and fall prevention behavior of patients, as well as the burden of falling, fall knowledge, interaction satisfaction, and fall prevention behavior of nurses. A paired *t*-test assessed whether post-test changes in the outcome variables regarding patients and nurses were significant relative to pre-test values.

#### 2.2.5. Ethical Considerations

This study was conducted for eight weeks after approval from the Institutional Review Board, Korea University (KUIRB-2019-0159-01) in Seoul, South Korea. The author received the clinical trial number (KCT0005908) from the Clinical Research Information Service in Seoul, South Korea. Prior to the start of the program, the objectives and procedures of the study were explained to the participants, and both oral and written consent were obtained. Small compensation was provided to the study participants. Consent was obtained from patients, legal guardians, and nurses in both control and intervention groups, and written consent included information on the purpose, necessity, expected effects, participation period, study procedure, and emergency response methods for vulnerable participants. Moreover, the consent noted that the collected data would not be used for purposes other than the study, and the participants would be coded in numbers for confidentiality. Moreover, the participants were informed that the study could be withdrawn at any time. In the control group, information regarding the fall prevention program was provided after the study was completed, and a fall prevention program was offered to those participants who wished to participate. All tools in this study were used after obtaining approval from the original authors.

## 3. Results

### 3.1. General and Disease-Related Characteristics

#### 3.1.1. Patients

There were 27 and 30 patients in the control and intervention groups, respectively. Among the general and disease-related characteristics of patients, there was a significant difference only in the informative past education programs. However, the number of patients with education experience was too small, and there were no significant differences between the groups for other variables (Table 2).

#### 3.1.2. Nurses

There were 28 and 30 nurses in the intervention and control groups, respectively. There were no significant differences between the groups, except for the age and falling experience of the assigned patients (Table 2).

### 3.2. Pre-Test Homogeneity Test for Study Variables

#### 3.2.1. Patients

The number of falls, fall prevention behavior, interaction satisfaction, fall knowledge, and fear of falling did not significantly differ between the two groups of patients (Table 2).

#### 3.2.2. Nurses

Fall prevention behavior, interaction satisfaction, fall knowledge, and burden of falling were not significantly different between the two groups of nurses (Table 2).

### 3.3. Effects of the Fall Prevention Program 

#### 3.3.1. Patients’ Number of Falls, Fall Prevention Behavior, Fall Knowledge, and Fear of Falling

The number of falls decreased from three to two after the intervention. In the control group, the number of falls increased from three to five; however, the number of falls was not significantly lower in the intervention group than in the control group (Z = −0.98, *p* = 0.326).

The mean fall prevention behavior increased from 2.38 to 3.83 after the intervention in the intervention group. The control group saw a 2.60 to 2.38 decrease. Thus, fall prevention behavior in the intervention group significantly increased relative to that in the control group (*t* = −11.66, *p* < 0.001). Fall knowledge increased from 6.89% to 13.41% after the intervention. The control group saw a slight increase from 8.13 to 8.27. Thus, fall knowledge in the intervention group significantly increased relative to that in the control group (*t* = −6.57, *p* < 0.001).

Fear of falling decreased from 6.34 to 1.91 after the intervention. In the control group, the fear of falling decreased from 6.34 to 5.91. Thus, the reduction was significant in the intervention group relative to the control group (*t* = 5.58, *p* < 0.001) (Table 3).

#### 3.3.2. Nurses’ Fall Prevention Behavior, Fall Knowledge, and Burden of Falling

Fall prevention behavior increased from a mean of 3.96 to 4.69 after the intervention. In the control group, it decreased from 3.78 to 3.76, and the fall prevention behavior of the intervention group significantly increased relative to that in the control group (*t* = −3.60, *p* < 0.001).

Fall knowledge increased from a mean of 13.50 to 15.79 after the intervention. It also increased from 13.43 to 14.00 in the control group. Thus, fall knowledge significantly increased in the intervention group relative to that in the control group (*t* = −2.67, *p* < 0.010). The burden of falling decreased from 2.76 to 2.70 after the intervention. In the control group, it increased from 2.69 to 2.84. Thus, the burden of falling did not significantly decrease in the intervention group relative to that in the control group (*t* = 1.65, *p* = 0.105) (Table 3).

#### 3.3.3. Interaction Satisfaction among Patients and Nurses

Interaction satisfaction in the intervention group of patients increased from 3.35 to 4.87 after the program. In the control group, it decreased from 3.43 to 3.32. Thus, the interaction satisfaction in the intervention group significantly increased relative to that in the control group (*t* = −8.06, *p* < 0.001) (Table 3).

In the intervention group of nurses, interaction satisfaction increased from 3.37 to 4.28 after the program. In the control group, it increased from 3.19 to 3.22. Thus, interaction satisfaction significantly increased in the intervention group relative to that in the control group (*t* = −4.16, *p* < 0.001) (Table 3).

## 4. Discussion

### 4.1. Fall Prevention Program Based on King’s Goal Achievement Theory

In this study, a fall prevention program based on King’s goal attainment theory was provided to elderly patients and nurses in a long-term care hospital to decrease the fear of falling, reduce the burden of falling of nurses, and increase fall knowledge and interaction satisfaction of both patients and nurses, ultimately increasing fall prevention behavior and reducing the number of falls. The program is significant in the following ways.

First, this study developed and applied a fall prevention program based on King’s goal attainment theory, in which patients and nurses participated together. Patient participation is regarded as an international standard for the healthcare system and the legal rights of patients. Thus, patients should participate in decisions related to health management planning, effects, and evaluations. In particular, patient-centered health care must be planned per the opinions, needs, and preferences of patients to allow them to maintain control over their health [53]. However, studies have shown that patients believe nurses are solely responsible for preventing falls and that their role in preventing falls is passive [54]. Therefore, patient-centered nursing [55] and interaction via communication are effective for treatment [56]. Both patient– and nurse–researcher and patient–nurse interactions were included in the fall prevention program based on King’s goal attainment theory to promote patient participation. Thus, fall prevention behavior, interaction satisfaction, and fall knowledge increased, while fear of falling among patients was reduced.

Second, a fall prevention program was developed and applied using guidelines and prints made of visual data of hospital conditions familiar to the patients. An effective fall prevention program requires environmental, educational, nursing process, and fall prevention interventions [57] per individual circumstances via effective communication [58]. Therefore, guidelines and prints containing visual data of familiar hospital conditions based on King’s goal attainment theory were used to provide systematic fall prevention education tailored to each patient. Additionally, this study included various meaningful contents, such as an environment-related fall prevention checklist, demonstrations, and practical education by rehabilitation therapists.

### 4.2. Effects of the Fall Prevention Program 

Fear of falling was reduced, and fall knowledge, interaction satisfaction, and fall prevention behavior increased regarding both patients and nurses. The results on the effects of the fall prevention program are discussed as follows.

Most studies have reported on knowledge [52], fall prevention behavior [24], and the number of falls [27]. However, no studies have examined the perception of falls and interactions. Moreover, fall prevention programs that included individual education tailored to each subject, demonstration, and repetitive education were challenging to find. Therefore, this study reduced the negative perception of falls by analyzing the fear of falling among patients and the burden of falling among nurses after the fall prevention program. Furthermore, interactions between patients and nurses were included to show that nurses are not solely responsible for falls and that fall prevention behaviors are more effective when both patients and nurses exercise such behavior.

In this study, nurses better understood the risk of falling and answered patient questions related to falls. Such an interaction allowed patients and nurses to continue fall prevention behaviors even beyond the study. Rehabilitation therapists and nurses in charge of patient safety participated in providing demonstrations and opportunities to practice necessary clinical skills. Further, education and demonstrations, exchange of opinions, counseling and support, and environmental management were included in the program via personal, interpersonal, and social systems, leading to a significant increase in fall prevention behavior.

Tzeng and Yin [59] suggested that understanding patient-centered care and patient involvement in fall prevention programs is necessary to prevent falls. In this study, patients and nurses set mutual goals together; the demands and needs of patients were reflected in the program. A grape sticker was provided to the patients every week when falls did not occur to induce confidence. The nurses were asked to encourage the patients to increase the effects of the program further.

A checklist, fall risk assessment tool, fall environment assessment tool, fall prevention patient education and re-education, fall report form, and hourly rounding have been suggested as effective for fall prevention [28]. In our study, a checklist, fall risk assessment, environmental assessment, repetitive individual education, fall reports, and periodic rounding were included in the program to promote fall prevention behaviors further.

In this study, the number of falls in the intervention group decreased from three to two (*p* = 0.480). In the control group, it increased from three to five (*p* = 0.705). Intuitively, the number of falls would decrease as the fall prevention behavior of patients improves. However, the number of falls was insufficient to observe a statistically significant difference, and the eight-week intervention period was insufficient relative to the 6-month fall intervention study that reported an effective decrease in the number of falls (*p* < 0.004) [60,61,62] and falls with injury (*p* < 0.01) [63]. Therefore, more participants should be selected in future studies, and the effects should be observed over a longer period of at least six months.

Further, fear of falling was lower in the patient intervention group than in the control group (*p* < 0.001). Expectedly, mutual goal setting and communication help assess objective and subjective risk factors of falling, thus leading to a decreased fear of falling. In particular, patient behavior characteristics were evaluated, and patients were encouraged to continue fall prevention activities to be actively involved in managing their health [64]. This study observed that patients were afraid of falling when traveling to bathrooms. Lim et al. [54] also reported that the number of falls was highest in bathrooms. During the intervention period of this study, two cases of falls were observed in the bathroom of the same patient.

Regarding the fear of falling, McMahon, Talley, and Wyman [65] reported that autonomy and independence of patient behavior are necessary. Therefore, in this study, patients were sufficiently educated on the risk of falling, and lighting was increased in surrounding environments to prevent as much falling as possible. Further, patients wore gait belts and were accompanied by a caregiver, and repetitive education was provided using an emergency bell for the toilet. Thus, cases of participants collapsing to the floor were observed. Given the likelihood of falls, opinions were exchanged between patients and staff to prevent continuous falls.

This study also aimed to lower the burden of falling among nurses; however, the effects were not significant (*p* = 0.105). This finding may have been due to nurses’ increased responsibility for patients’ fall accidents and the increased burden of work [66]. In most medical negligence claims, nurses are often responsible. Thus, falls are an important problem for nurses [67]. Long-term care hospital patients are elderly and suffer from complex chronic diseases [68]; confusion, gait problems, Alzheimer’s, loss of directional senses, and failure to comply with safety guidelines are frequently observed risk factors [59]. Therefore, continuous education and evaluation, activities suitable for nursing manpower structures, and systematic management are required [69].

Particularly, it is necessary to develop educational programs to help nurses overcome the effects of falls, prevent falls, and improve patient safety [70]. The Patient Safety Act, effective since July 2016, states that medical staff are required to voluntarily report patient safety incidents to avoid the omission of reporting due to fear or guilt of punishment and that adverse actions cannot be taken against the one who reported, given the aim of the report [71]. Thus, the hospital environment that considers falls as the full responsibility of nurses should be reviewed; emotional and educational support must be provided to reduce the burden of falling among nurses. Moreover, an improved legal system should be established to avoid imposing excessive responsibility on nurses.

In this study, pictures of high-risk hospital situations were incorporated into brochures and prints for individual education on fall knowledge. As most of the patients answered that they were not aware of their medications, medication description sheets with photos and explanations were printed and explained by assigned nurses. Moreover, explanations were repeated by the researchers to ensure comprehension. Lim et al. [54] reported that fall prevention programs provided to all patients during patient waiting time at hospitals are not effective. In our study, fall prevention education was provided to every patient at the time of hospitalization; however, only a few patients were aware of such education. Therefore, this finding suggests that additional fall prevention education is required for patients.

Personal attention and effort are important for elderly patients. However, personal effort alone cannot prevent falls due to old age, disease status, and medication difficulties [4]. Thus, the education program must be modified regularly per changing fall knowledge and tasks (*p* = 0.001) [72]. Further, the appropriate methods, quantity, and education intensity must be provided per the knowledge level of elderly patients (*p* < 0.001) [9], and efforts such as systematic education and evaluation are required [73].

Altogether, as elderly and vulnerable patients are mostly in long-term care hospitals, a fall prevention program suitable for long-term care hospitals is needed. Therefore, in this study, fall knowledge, diseases, and medications for each elderly patient were identified. Repetitive education was individually provided and fall prevention behaviors were improved through the interaction between patients and nurses, who were aware of the health status and lifestyle of patients. Hence, the fall prevention program based on King’s goal attainment theory is relevant in that it significantly reduces the fear of falling among patients and improves fall knowledge, interaction satisfaction, and fall prevention behavior of patients and nurses through transactions, including systems and interactions between patients and nurses. This study provides a basis for developing a systematic fall prevention system and positively contributing to fall prevention in practice.

### 4.3. Limitations

First, the study design and sampling methods were not randomly assigned. The separation of floors between wards was performed in a non-equivalent control pre- and post-test study. Moreover, this study was conducted at a single hospital in Seoul, and the results cannot be generalized to patients and nurses in all long-term care hospitals. Therefore, future studies should employ a fall prevention program using a cluster or subject randomization method for several long-term care hospitals. Second, only short-term effects immediately before the intervention and eight weeks of the intervention period were assessed. The participants were not followed up, and long-term continuous effects of the program could not be assessed. Thus, it is necessary to assess the continuous effects of the intervention in future studies. Third, there was no significant difference in terms of prior homogeneity between groups, and since the data did not follow normality and proceeded to non-parametric statistics, the corresponding variable was not adjusted. However, if sufficient samples are collected and various characteristic variables are corrected, more clear effect verification will be possible in future studies. Fourth, fear of falling decreased, and fall knowledge, interaction satisfaction, and fall prevention behavior improved among patients in this study; however, the number of falls and burden of falling on nurses did not significantly change. Therefore, further studies are needed to reduce the number of falls and the burden of falling among nurses.

## 5. Conclusions

In this study, a fall prevention program was developed based on King’s goal attainment theory, and the effects of the developed program were assessed. The fall prevention program employed transactions of personal systems (fear of falling, burden of falling, and fall knowledge), interpersonal system (interaction satisfaction), and social system (fall prevention behavior). Thus, fear of falling was reduced among patients, and fall knowledge, interaction satisfaction, and fall prevention behavior of patients were improved. Although the burden of falling among nurses was not reduced statistically, fall knowledge, interaction satisfaction, and fall prevention behavior of nurses were improved. Therefore, based on the positive effects identified in this study, it is thought that the fall prevention program based on the goal attainment theory will be applied to clinical studies and research to help prevent falls.

## Figures and Tables

**Table 1:** Basic principles and implementation schedule of the program based on King’s goal attainment theory.

King’s Conceptual System	King’s Concept	Configuration Element	Main Strategy	Goal	Intervention Content			Intervention Methods			
								Individual Education	Group Education	Individual Counseling	Individual Activities
Personal system	Perception	Fear of falling	Problem assessment Knowledge and information provision	Decreased fear of falling among patients	(1) Assessment of problems related to falling among patients (2) Understanding the fear of falling			●		●	
		Burden of falling	Problem assessment Knowledge and information provision	Reduced burden of falling among nurses	(1) Assessment of problems related to falling regarding nurses (2) Understanding the burden of falling			●		●	
	Growth and development	Fall knowledge	Knowledge and information provision	Improved fall knowledge	(1) Understanding fall knowledge using guidelines and prints			●	●		
Interpersonal system	Communication	Interaction satisfaction	Goal setting and motivation Knowledge and information provision	Increased fall prevention behavior through communication Increased interaction satisfaction	(1) Mutual goal setting to reduce the number of falls (2) Assessment of disturbance factors for fall prevention (3) Education on fall prevention guidelines and demonstrations (4) Feedback on understanding after education (5) Mutual assessment of fall prevention checklist (6) Fall prevention education using the 5A method by the assigned nurse 7) Medication education			●	●		●
	Interaction										
Social System	Education system	Fall prevention behavior	Improved function and behavior	Improved fall prevention behavior	(1) Fall prevention education for patients and nurses (2) Fall prevention education with therapists and a nurse in charge of patient safety			●	●		
	Social support		Motivation and emotional support	Enhanced motivation through improved social support	(1) Consultation and support for difficulties and concerns related to falls among patients (2) Consultation and support for difficulties and concerns related to falls regarding nurses (3) Supporting continued participation for fall prevention					●	
**Group**		**Intervention Method**		**Program Schedule (Weeks)**							
				**1**	**2**	**3**	**4**	**5**	**6**	**7**	**8**
Intervention group		Individual education		●	●	●	●	●	●	●	●
		Group education			●						
		Individual counseling		●	●	●	●	●	●	●	●
		Individual activities						●	●		

**Table 2:** Pre-test homogeneity test of general and disease-related characteristics between patients and nurses in the control and intervention groups.

Variable	Classification	Patient Intervention Group (*n* = 27) Mean ± SD or Number (%) or Cases	Patient Control Group (*n* = 30) Mean ± SD or Number (%) or Cases	Total	χ^2^/ Z/ t	*p*
Age		78.78 ± 9.50	78.77 ± 10.58	78.77 ± 9.99	0.00	0.997
Number of days in the hospital		583.52 ± 444.28	376.60 ± 349.08	474.61 ± 406.95	1.97	0.054
Sex	Female	18 (66.7)	19 (63.3)	37 (64.9)	0.07	0.792
	Male	9 (33.3)	11 (36.7)	20 (35.1)		
Educational level	Elementary school	6 (22.2)	8 (26.7)	14 (24.6)	0.62	0.961
	Middle school	5 (18.5)	4 (13.3)	9 (15.8)		
	High school	5 (18.5)	5 (16.7)	10 (17.5)		
	Professional school	1 (3.7)	2 (6.7)	3 (5.3)		
	College and above	10 (37.0)	11 (36.7)	21 (36.8)		
Fall experience in the past year	Yes	5 (18.5%)	8 (26.7%)	13 (22.8%)	0.66	0.513
	No	22 (81.5%)	22 (73.3%)	44 (71.2%)		
Experience of fall prevention education in the past year	Yes	5 (18.5)	6 (20.0)	11 (19.3)	0.02	0.887
	No	22 (81.5)	24 (80.0)	46 (80.7)		
How informative was the education session	Very helpful	0 (0.0)	5 (83.3)	5 (45.5)	7.77	0.026
	Helpful	4 (80.0)	1 (16.7)	5 (45.5)		
	Not helpful	1 (20.0)	0 (0.0)	1(9.1)		
Diagnosis	Cerebrovascular disease	22 (81.5)	26 (86.7)	48 (84.2)	0.29	0.592
	Parkinson’s disease	3 (11.1)	3 (10.0)	6 (10.5)	0.02	0.891
	Dementia	15 (55.6)	23 (76.7)	38 (66.7)	2.85	0.091
	Femur fracture	0 (0.0)	1 (3.3)	1 (1.8)	0.92	0.339
	Others	3 (11.1)	0 (0.0)	3 (5.3)	3.52	0.061
Comorbidity	Hypertension	22 (81.5)	23 (76.7)	45 (78.9)	0.20	0.656
	Diabetes	5 (18.5)	6 (20.0)	11 (19.3)	0.02	0.887
	Cerebrovascular disease	24 (88.9)	27 (90.0)	51 (89.5)	0.02	0.891
	Parkinson’s disease	4 (14.8)	4 (13.3)	8 (14.0)	0.02	0.872
	Hemiparalysis	22 (81.5)	24 (80.0)	46 (80.7)	0.02	0.887
	Paraplegia	3 (11.1)	0 (0.0)	3 (5.3)	3.52	0.061
Anti-psychotic medication	Yes	9 (33.3)	14 (46.7)	23 (40.4)	1.05	0.306
	No	18 (66.7)	16 (53.3)	34 (59.6)		
MMSE		24.44 ± 4.10	23.97 ± 3.84	24.19 ± 3.93	0.46	0.651
MFS		50.19 ± 23.88	56.33 ± 21.49	53.42 ± 22.66	1.02	0.331
Variable	Classification	Nurse intervention group (*n* = 28) Mean ± SD or number (%)	Nurse control group (*n* = 30) Mean ± SD or number (%)	Total	χ^2^/t	*p*
Age		42.25 ± 7.53	35.97 ± 9.83	39.00 ± 9.28	2.72	0.008
Sex	Female	28 (100.0)	27 (90.0)	55 (94.8)	2.95	0.086
	Male	0 (0.0)	3 (10.0)	3 (5.2)		
Educational level	Professional school	11 (39.3)	9 (30.0)	20 (34.5)	0.55	0.457
	College	17 (60.7)	21 (70.0)	38 (65.5)		
Working experience		11.80 ± 8.07	8.01 ± 6.22	9.84 ± 7.36	2.01	0.052
Fall experience of assigned patients in the past year	Yes	18 (64.3)	26 (86.7)	44 (75.9)	3.96	0.047
	No	10 (35.7)	4 (13.3)	14 (24.1)		
Fall prevention education in the past year	Yes	28 (100.0)	29 (96.7)	57 (98.3)	0.95	0.330
	No	0 (0.0)	1 (3.3)	1 (1.7)		
How informative was the education session	Very helpful	11 (39.3)	5 (17.2)	16 (28.1)	4.25	0.119
	Helpful	15 (53.6)	23 (79.3)	38 (66.7)		
	Not helpful	2 (7.1)	1 (3.4)	3 (5.3)		
Nursing performance for fall prevention	Strongly agree	7 (25.0)	3 (10.0)	10 (17.2)	3.12	0.374
	Agree	19 (67.9)	24 (80.0)	43 (74.1)		
	Disagree	2 (7.1)	2 (6.7)	4 (6.9)		
	Strongly disagree	0 (0.0)	1 (3.3)	1 (1.7)		
Necessity of fall prevention education	Strongly agree	12 (42.9)	12 (40.0)	24 (41.4)	0.05	0.825
	Agree	16 (57.1)	18 (60.0)	34 (58.6)		

**Table 3:** Pre–post-difference test of the main study variables between patients and nurses in the control and intervention groups.

Variable	Time	Patient Intervention Group (*n* = 27)	Patient Control Group (*n* = 30)	Z/t	*p*
		N (%) or Cases	N (%) or Cases		
Number of falls	Pre-test	3	3	−0.14	0.892
	Post-test	2	5	−1.22	0.222
	Pre-post difference	−1	2	−0.98	0.326
	*Z*(*p*)	−0.71 (0.480)	−0.38 (0.705)		
Fall prevention behavior	Pre-test	2.38 ± 0.42	2.60 ± 0.60	1.57	0.121
	Post-test	3.83 ± 0.22	2.38 ± 0.55	−13.25	<0.001
	Pre-post difference	1.44 ± 0.49	−0.22 ± 0.58	−11.66	<0.001
	*t*(*p*)	−15.26 (<0.001)	2.12 (0.043)		
Fall knowledge	Pre-test	6.89 ± 3.19	8.13 ± 3.41	1.42	0.162
	Post-test	13.41 ± 1.72	8.27 ± 4.39	−5.93	<0.001
	Pre-post difference	6.52 ± 3.57	0.13 ± 3.79	−6.57	<0.001
	*t*(*p*)	−9.50 (<0.001)	−0.20 (0.847)		
Fear of falling	Pre-test	6.34 ± 1.87	6.34 ± 2.17	−0.01	0.994
	Post-test	1.91 ± 1.23	5.91 ± 2.95	6.79	<0.001
	Pre-post difference	−4.43 ± 2.27	−0.43 ± 3.04	5.58	<0.001
	*t*(*p*)	10.15 (<0.001)	0.78 (0.444)		
Interaction satisfaction	Pre-test	3.35 ± 0.54	3.43 ± 0.49	0.59	0.558
	Post-test	4.87 ± 0.42	3.32 ± 0.61	−11.33	<0.001
	Pre-post difference	1.52 ± 0.69	−0.11 ± 0.82	−8.06	<0.001
	*t*(*p*)	−11.39 (<0.001)	0.74 (0.465)		
Variable	Time	Nurse intervention group (*n* = 28)	Nurse control group (*n* = 30)	t	*p*
		Mean ± SD	Mean ± SD		
Fall prevention behavior	Pre-test	3.96 ± 0.48	3.78 ± 0.74	−1.12	0.269
	Post-test	4.69 ± 0.35	3.76 ± 0.71	−6.40	<0.001
	Pre-post difference	0.73 ± 0.68	−0.02 ± 0.88	−3.60	<0.001
	*t*(*p*)	−5.67 (<0.001)	0.11 (0.912)		
Fall knowledge	Pre-test	13.50 ± 2.43	13.43 ± 2.11	−0.11	0.911
	Post-test	15.79 ± 0.63	14.00 ± 1.64	−5.54	<0.001
	Pre-post difference	2.29 ± 2.46	0.57 ± 2.43	−2.67	0.010
	*t*(*p*)	−4.91 (<0.001)	−1.28 (0.212)		
Burden of falling	Pre-test	2.76 ± 0.26	2.69 ± 0.26	−0.92	0.362
	Post-test	2.70 ± 0.51	2.84 ± 0.48	1.03	0.306
	Pre-post difference	−0.05 ± 0.49	0.14 ± 0.43	1.65	0.105
	*t*(*p*)	0.58 (0.565)	−1.85 (0.074)		
Interaction satisfaction	Pre-test	3.37 ± 0.70	3.19 ± 0.54	−1.10	0.278
	Post-test	4.28 ± 0.67	3.22 ± 0.67	−6.45	<0.001
	Pre-post difference	0.92 ± 0.84	0.04 ± 0.77	−4.16	<0.001
	*t*(*p*)	−5.78 (<0.001)	−0.26 (0.795)		

## Data Availability

All data generated or analyzed during this study are included in this published article.

## References

[1] Kostat Elderly. https://kostat.go.kr/portal/korea/kor_nw/1/1/index.board?bmode=read&bSeq=&aSeq=377701&pgeNo=1&rowNum=10&navCount=10&currPg=&searchInfo=srch&sTarget=title&sTxt=%EA%B3%A0%EB%A0%B9%EC%9E%90+%ED%86%B5%EA%B3%84.

[2] Kostat Special Estimates for Future Furniture. https://kostat.go.kr/portal/korea/kor_nw/1/2/1/index.board?bmode=read&bSeq=&aSeq=377540&pageNo=2&rowNum=10&navCount=10&currPg=&searchInfo=&sTarget=title&sTxt=.

[3] National Health Insurance Service (NHIS) (2019). Health Insurance Statistical Yearbook. https://www.nhis.or.kr/nhis/together/wbhaea01600m01.do?mode=view&articleNo=10802558&article.offset=80&artcleLimit=10.

[4] Miake-Lye I.M., Hempel S., Ganz D.A., Shekelle P.G. (2013). Inpatient fall prevention programs as a patient safety strategy: A systematic review. Ann. Intern. Med..

[5] Yoo K.S. (2017). Knowledge, attitude and prevention activities related to fall among of geriatric hospital nurse. J. Korean Public Health Nurs..

[6] Heung M., Adamowski T., Segal J.H., Malani P.N. (2010). A successful approach to fall prevention in an outpatient hemodialysis center. Clin. J. Am. Soc. Nephrol..

[7] Carroll D.L., Dykes P.C., Hurley A.C. (2010). Patients’ perspectives of falling while in an acute care hospital and suggestions for prevention. Appl. Nurs. Res..

[8] World Health Organization Falls. http://www.who.int/mediacentre/factsheets/fs344/en/.

[9] Chung M.S. (2013). The effects of fall prevention education on the fall-related knowledge and prevention activity of the elderly hospitalized in internal medicine department. J. Muscle Jt. Health.

[10] Bae J., Cho S.I. (2014). Effects of community-based comprehensive call prevention program on muscle strength, postural balance and fall efficacy in elderly people. J Korean Acad. Nurs..

[11] World Health Organization Global Report on Falls Prevention in Older Age. https://extranet.who.int/agefriendlyworld/global-report-on-falls-prevention-in-older-age/.

[12] Glogovsky D. (2017). How can policy change guide nursing practice to reduce in-patient falls?. Nursing2020.

[13] Lopez K.D., Gerling G.J., Cary M.P., Kanak M.F. (2010). Cognitive work analysis to evaluate the problem of patient falls in an inpatient setting. J. Am. Med. Inform. Assoc..

[14] Gu Y.Y., Balcaen K., Ni Y., Ampe J., Goffin J. (2016). Review on prevention of falls in hospital settings. Chin. Nurs. Res..

[15] Park Y.H. (2016). Characteristics and ADL (Activities of Daily Living) associated factors of elderly inpatients in long-term care hospitals: A survey of patients (2013–2014). Korean J. Health Serv. Manag..

[16] Lee Y.J., Gu M.O. (2015). The fall related circumstance, fall risk factor, and fall predictors of inpatients of small and medium sized hospitals. Clin. Nurs. Res..

[17] King B., Pecanac K., Krupp A., Liebzeit D., Mahoney J. (2018). Impact of fall prevention on nurses and care of fall risk patients. Gerontologist.

[18] Njine D., Soroka B. (2016). Good Quality Interaction between the Registered Nurse and the Patient: A Systematic Review. Degree Programme in Nursing.

[19] Jang H.K., Lee J.Y., Kim M.K., Yang E.O., Gil C.R. (2019). Reliability and validity of Korean version of the nurse-patient interaction scale. J. Korea Acad. Industr. Coop. Soc..

[20] King I.M. (1981). A Theory for Nursing Systems, Concepts, Process.

[21] de Leon-Demare K., MacDonald J., Gregory D.M., Katz A., Halas G. (2015). Articulating nurse practitioner practice using King’s theory of goal attainment. J. Am. Assoc. Nurs. Pract..

[22] Araújo E.S.S., Silva L.F.D., Moreira T.M.M., Almeida P.C.D., Freitas M.C.D., Guedes M.V.C. (2018). Nursing care to patients with diabetes based on King’s theory. Rev. Bras. Enferm..

[23] Shim S.M., Kim E.H. (2019). Effect of fall prevention education for older patients in comprehensive nursing care service ward. J. Korean Public Health Nurs..

[24] Kang Y.O., Song R.Y. (2018). Effects of fall prevention education program on attitu.des, prevention behaviors, and satisfaction among elderly inpatients. Korean J. Adult Nurs..

[25] Lee H.O., Lee B.H., Lee C.H. (2017). Effect of strength exercise on patient fall prevention program: Focusing on the fall high risk group elderly patients. J. Health Inform. Stat..

[26] Cho E.K., Sung M.H., Lee Y.S., Seok S.H. (2019). Effects of fall prevention educational program for nurses in comprehensive nursing care units. J. Korea Contents Assoc..

[27] Park B.M., Ryu H.S., Kwon K.E., Lee C.Y. (2019). Development and effects of a fall prevention program based on King’s goal attainment theory for fall high-risk elderly patients in long-term care hospital. J. Korean Acad. Nurs..

[28] Lee J.H., Kim H.A., Park S.W. (2015). Prevention of fall in the hospital. J. Korean Med. Assoc..

[29] Kim E.K., Lee J.C., Eom M.R. (2008). Fall risk factors of inpatients. J. Korean Acad. Nurs..

[30] Lee H.J. (2018). Identifying Characteristics of Fall Episodes and Fall-Related Risk Predictors. Master’s Thesis.

[31] Baek Y.J., Jung I.S. (2012). Fall risk of patients admitted to emergency room. Global Health Nur..

[32] Pohl P., Sandlund M., Ahlgren C., Bergvall-Kåreborn B., Lundin-Olsson L., Wikman A.M. (2015). Fall risk awareness and safety precautions taken by older community-dwelling women and men—A qualitative study using focus group discussions. PLoS ONE.

[33] Adib-Hajbaghery M., Tahmouresi M. (2018). Nurse–patient relationship based on the Imogene King’s theory of goal attainment. Nurs. Midwifery Stud..

[34] Ladee C., Lagampan S., Pichayapinyo P., Mayurasakorn K., Lagampan C. (2020). Effect of a goal attainment nursing program on self-management and blood pressure control in high-risk hypertensive patients in a primary care unit. Siriraj Med. J..

[35] Killeen M.B., King I.M. (2007). Use of King’s conceptual system, nursing informatics, and nursing classification systems for global communication. Int. J. Nurs. Terminol. Classif..

[36] Lee C.H. (2019). Development and Evaluation of Fall Prevention Program Based on King’s Goal Attainment Theory for the Elderly with Lower Extremity Osteoarthritis in the Community. Ph.D. Thesis.

[37] How-to Guide: Reducing Patient Injuries from Falls. https://www.ihi.org/resources/Pages/Tools/TCABHowToGuideReducingPatientInjuriesfromFalls.aspx.

[38] Korea Institute for Healthcare Accreditation (KOIHA) 3rd Period Long Term Care Hospital Certification Standards Promulgated. https://www.koiha.or.kr/member/kr/board/establish/establish_BoardList.do.

[39] Ministry of Health and Welfare (MOHW) The First Comprehensive Patient Safety Plan. https://www.mohw.go.kr/react/jb/sjb030301vw.jsp?PAR_MENU_ID=03&MENU_ID=0319&CONT_SEQ=344873&page=1.

[40] The Clinical Practice Guideline Treating Tobacco Use and Dependence 2008 Update Panel, Liaisons, and Staff (2008). A clinical practice guideline for treating tobacco use and dependence: 2008 update: A U.S. public health service report. Am. J. Prev. Med..

[41] Krauss M.J., Tutlam N., Costantinou E., Johnson S., Jackson D., Fraser V.J. (2008). Intervention to prevent falls on the medical service in a teaching hospital. Infect. Control Hosp. Epidemiol..

[42] Faul F., Erdfelder E., Buchner A., Lang A.G. (2009). Statistical power analyses using G*power 3.1: Tests for correlation and regression analyses. Behav. Res. Methods.

[43] Jung I.J., Kim S.J. (2017). Effects of group counseling program based on King’s goal attainment theory for middle school students with emotional and behavioral problems. J. Korean Acad. Nurs..

[44] Tinetti M.E., Richman D., Powell L. (1990). Falls efficacy as a measure of fear of falling. J. Gerontol..

[45] Jang J.M. (2005). A Structural Model for Falls and Quality of Life in Elderly People Living at Home. Ph.D. Thesis.

[46] Kim M.Y. (2014). A Study of Fall Risk, Fear of Falling, and Depression in Patients after Brain Tumor Surgery. Master’s Thesis.

[47] Kim C.K. (2002). An Analysis of Fall Incidence Rate and Its Related Factors of Fall in in Patients. Ph.D. Thesis.

[48] Kim M.Y. (2008). Fall-Related Knowledge and Prevention Behavior among Hospitalized Elderly Inpatients. Master’s Thesis.

[49] Lim S.R., Kwon J.H. (1998). Marital communication behavior and marital satisfaction. Korean J. Clin. Psychol..

[50] Kim Y.J. (2010). Nurse-patient interaction patterns and patient satisfaction in the emergency department. J. Korean Acad. Nurs..

[51] Kim M.S., Kim J.S. (2013). Effects of the fall prevention education program (FPEP) for caregivers in elderly care facilities on fall-related knowledge, fall-related burden, and caring behaviors for fall prevention. J. Korea Contents Assoc..

[52] Kim S.H., Seo J.M. (2017). Geriatric hospital nurses’ knowledge, attitude toward falls, and fall prevention activities. J. Korean Gerontol. Nurs..

[53] Vahdat S., Hamzehgardeshi L., Hessam S., Hamzehgardeshi Z. (2014). Patient involvement in health care decision making: A review. Iran Red. Crescent Med. J..

[54] Lim M.L., Ang S.G.M., Teo K.Y., Wee Y.H.C., Yee S.P., Lim S.H., Ang S.Y. (2018). Patients’ experience after a fall and their perceptions of fall prevention: A qualitative study. J. Nurs. Care Qual..

[55] Herbst A.M., Friesen M.A., Speroni K.G. (2013). Caring, connecting, and communicating: Reflections on developing a patient-centered bedside handoff. Int. J. Hum. Caring.

[56] Doane G.H., Varcoe C. (2007). Relational practice and nursing obligations. Adv. Nurs. Sci..

[57] Zhao Y.L., Bott M., He J., Kim H., Park S.H., Dunton N. (2019). Evidence on fall and injurious fall prevention interventions in acute care hospitals. J. Nurs. Adm..

[58] Yardley L., Donovan-Hall M., Francis K., Todd C. (2007). Attitudes and beliefs that predict older people’s intention to undertake strength and balance training. J. Gerontol. B Psychol. Sci. Soc. Sci..

[59] Tzeng H.M., Yin C.Y. (2015). Patient engagement in hospital fall prevention. Nurs. Econ..

[60] Dykes P.C., Carroll D.L., Hurley A., Lipsitz S., Benoit A., Chang F., Middleton B. (2010). Fall prevention in acute care hospitals: A randomized trial. J. Am. Med. Assoc..

[61] Fonda D., Cook J., Sandler V., Bailey M. (2006). Sustained reduction in serious fall-related injuries in older people in hospital. Med. J. Aust..

[62] Healey F., Monro A., Cockram A., Adams V., Heseltine D. (2004). Using targeted risk factor reduction to prevent falls in older in-patients: A randomised controlled trial. Age Ageing.

[63] Barry E., Laffoy M., Matthews E., Carey D. (2001). Preventing accidental falls among older people in long stay units. Ir. Med. J..

[64] Kiyoshi-Teo H., Northrup-Snyder K., Cohen D.J., Dieckmann N., Stoyles S., Winters-Stone K., Eckstrom E. (2019). Older hospital inpatients’ fall risk factors, perceptions, and daily activities to prevent falling. Geriatr. Nurs..

[65] McMahon S., Talley K.M., Wyman J.F. (2011). Older people’s perspectives on fall risk and fall prevention programs: A literature review. Int. J. Older People Nurs..

[66] Spoelstra S.L., Given B.A., Given C.W. (2012). Fall prevention in hospitals: An integrative review. Clin. Nurs. Res..

[67] Kim C.G. (2011). Nurses’ knowledge and attitude toward fall in hospitalized patients. J. Ind. Sci. Res..

[68] Kim H.J., Kim H.Y. (2016). Experience of job stress among nurses working in long-term care hospital: A phenomenological approach. J. Korean Acad. Adult Nurs..

[69] Ryu J.M., Kim M.S. (2016). Influence of professional self-concept, self-leadership on elderly care performance of geriatric hospital nurses. J. Health Inform. Stat..

[70] Kim Y.J. (2017). Nurses’ experience of inpatients’ falls. J. Korean Acad. Fundam. Nurs..

[71] Korea Ministry of Government Legislation (KMGL) Patient Safety Act. https://www.law.go.kr/LSW/lsLinkProc.do?&lsNm=%ED%99%98%EC%9E%90%EC%95%88%EC%A0%84%EB%B2%95&chrClsCd=010202&mode=20&ancYnChk=0#.

[72] Abou El Enein N.Y., Abd El Ghany A.S., Zaghloul A.A. (2012). Knowledge and performance among nurses before and after a training programme on patient falls. Open J. Nurs..

[73] Jung J.Y., Jung K.H. (2016). The affect factors of geriatric hospital nurse’s falls prevention activities. J. Health Inform. Stat..

